# Circadian light

**DOI:** 10.1186/1740-3391-8-2

**Published:** 2010-02-13

**Authors:** Mark S Rea, Mariana G Figueiro, Andrew Bierman, John D Bullough

**Affiliations:** 1 Lighting Research Center, Rensselaer Polytechnic Institute, 21 Union Street, Troy, NY 12180, USA

## Abstract

The present paper reflects a work in progress toward a definition of circadian light, one that should be informed by the thoughtful, century-old evolution of our present definition of light as a stimulus for the human visual system. This work in progress is based upon the functional relationship between optical radiation and its effects on nocturnal melatonin suppression, in large part because the basic data are available in the literature. Discussed here are the fundamental differences between responses by the visual and circadian systems to optical radiation. Brief reviews of photometry, colorimetry, and brightness perception are presented as a foundation for the discussion of circadian light. Finally, circadian light (CL_A_) and circadian stimulus (CS) calculation procedures based on a published mathematical model of human circadian phototransduction are presented with an example.

## Introduction

The suprachiasmatic nuclei (SCN) in the hypothalamus host the master circadian clock that organizes and orchestrates the timing of all daily biological functions, from complicated physiological systems to single cells. The SCN in humans have, on average, an intrinsic period slightly greater than 24 hours [1] that is modulated by the temporal pattern of light and dark on the retina. As a result of the earth's rotation on its axis, the temporal pattern of light and dark on the retina synchronizes the SCN to a matching 24-h period. Recent research has demonstrated that disruption of the natural, 24-h pattern of light and dark from rapid flight across time zones or from rotating shift work can lead to a wide variety of maladies, from poor performance to sleep loss, weight gain, and even breast cancer [2-9]. Because it is increasingly evident that retinal light and dark exposures can profoundly affect human health and well-being, it is increasingly important to be able to quantify both light and dark as stimuli to the human circadian system.

The present paper deals with the evolving definition of circadian light. Technically, the adjective *circadian *must be used to modify the noun *light *because light is defined specifically in terms of optical radiation capable of producing a visual sensation in humans [10,11]. Strictly speaking then, light cannot be used synonymously with optical radiation capable of producing a non-visual, circadian response in humans or with optical radiation producing a visual response in another species. Nevertheless, in the vernacular, light is used as a term to describe optical radiation with a spectral power distribution anywhere within the "visible region" of the electromagnetic spectrum (approximately 380 nm to 730 nm), irrespective of its biological consequences. Moreover, the term light is always used, with or without strict regard for its ability to stimulate human vision, as a noun to describe *the stimulus to *rather than *the response from *a biological system. This is an important point because light is circularly defined; light as a stimulus to the human visual system was derived from responses by the human visual system. Thus, any formal definition of circadian light as a stimulus to the circadian system must also be dependent on the measured response from the circadian system. Fundamentally then, it is necessary to be able to measure a reliable response of the human circadian system to optical radiation incident on the retina to define the stimulus to the human circadian system. This inherent, and potentially confusing, circularity always must be considered as a formal definition of circadian light develops.

Notwithstanding this potentially confusing circularity, it will be difficult to develop a definition of light for the circadian system that is strictly homologous with the formal definition of light for the visual system because, for reasons discussed in this paper, the responses by these two systems to optical radiation on the retina are fundamentally different. The biophysical mechanisms underlying phototransduction for the two systems are similar but different enough to require thoughtful deliberation as a definition of circadian light evolves. Without a clear understanding of these differences, experimental results from studies of the impact of optical radiation on circadian physiology can be easily misinterpreted. Since, however, so much history and thought underlie our concept of light based upon the human visual system, these insights make the discussion of circadian light more readily explained and more easily understood. For this reason brief reviews of photometry, colorimetry and brightness perception are presented as a foundation for the discussion of circadian light.

## The photopic luminous efficiency function

**Figure 1:**
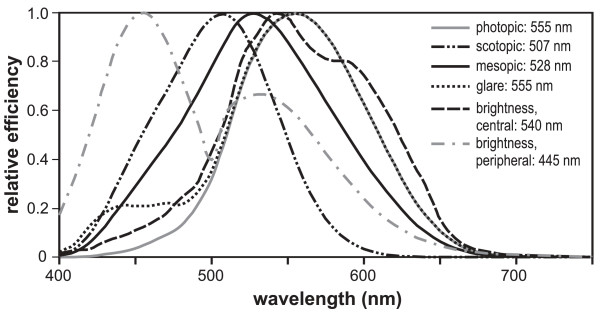
**Photopic and scotopic luminous efficiency functions **[10]** and other spectral sensitivity functions measured with humans (mesopic: Rea et al**. [13], **glare: Bullough **[14], **brightness, central: CIE **[10], **brightness, peripheral: Weale **[15]**)**. Peak wavelengths for each function are noted in the legend.

Psychophysical experiments were conducted by several laboratories nearly a century ago to develop "the spectral sensitivity of human vision." Following a consensus process, the data from these experiments were combined to form V_λ_, the photopic luminous efficiency function formally defining *light *[12], shown in Figure 1. V_λ _then is the bridge between radiometry, the measurement of radiant energy, and photometry, the measurement of light. Depending upon the geometric properties of interest, radiant flux (radiant energy per unit time) is weighted by V_λ _in the fundamental definitions of *luminous intensity *(V_λ_-weighted radiant intensity, or radiant flux within a solid angle), *illuminance *(V_λ_-weighted irradiance, or radiant flux incident on a surface area), and *luminance *(V_λ_-weighted radiance, or luminous intensity per unit area of a surface) [11]. Circadian light could then similarly bridge radiometry to circadian photometry and would have parallel definitions with those used for light based upon geometrical considerations.

The photopic luminous efficiency function is actually only one of a wide variety of functions that can be used to characterize the spectral sensitivity of the human visual system. Figure 1 also shows a small sample of human spectral sensitivity functions published in the literature [12-15]. In fact, depending upon the experimental conditions, *many *spectral response functions can be obtained from the human visual system. V_λ _is quite special, however, because, in addition to its metrological seniority, it has the important practical feature of exhibiting additivity. Additivity means that when two lights (A and B) of different spectral power distributions but of equal luminance (A = B) are combined by unit fractional amounts, they will continue to have the same luminance [13]. That is,(1)

where p and q are unit fractional amounts, such that p + q = 1

Additivity as defined by Equation 1 significantly restricts the relevance of V_λ _for representing the spectral sensitivity of the human visual system to a small handful of visual task conditions [16]. Because of additivity, however, V_λ _has become the universal "visual response function" for commerce and for government regulations [11].

Despite its assumed universality, the psychophysical techniques used to develop V_λ _only functionally characterize the spectral sensitivity of the achromatic (luminance) channel for the human fovea which is dominated by input from only two of the three cone types. The fovea constitutes only about 2% of the retina and provides humans with high spatial resolution. Only densely packed long-wavelength (L) and middle-wavelength (M) sensitive cones are found in the center of the fovea; although all three cone types are found throughout the remainder of the retina, the short-wavelength (S) sensitive cones are much rarer and, like the rods, absent from the center of the fovea. The S cone is also slower to respond to rapid modulations of light level than the L and M cones [17]. V_λ _is largely (but not exclusively) based upon a psychophysical technique known as flicker photometry. A small disc presented to an observer at the center of the fovea oscillates in time (flickers) between two lights of different spectral power distributions (perceived colors). By gradually adjusting the radiance of one light and the flicker rate, the two lights eventually appear as a steady light of a single hue. At this point where the two oscillating lights just fuse into what appears to be a fixed luminous disc, the two lights are defined as having the same luminance. V_λ _is determined by taking the reciprocal of the radiance at each wavelength needed to reach this constant-luminance flicker criterion and normalizing these values to the reciprocal of the radiance associated with the wavelength requiring the least amount of optical radiation needed to elicit the criterion response (λ_max _= 555 nm). By utilizing rapidly oscillating lights in the fovea, the S cone is functionally excluded from the definition of light even though this photoreceptor plays an extremely important role in our perception of brightness [15]. Nevertheless, V_λ _has gained ubiquity in metrology because additivity is essential for any system of photometry supporting commerce and government.

To be useful, a system of photometry first must, more or less, describe the relative brightness of a light source. The current system of photometry based on V_λ _does so, more or less. As a "white" light source (e.g., daylight, incandescent, fluorescent) generates greater radiance, the light source should appear brighter and the photometric quantity should increase. Further, as the spectral power distribution of the light emitted by a source shifts to the middle of the "visible spectrum" (i.e., near 555 nm) the source should also appear brighter and the photometric quantity should also increase. In general, photometry based upon V_λ _provides quantities consistent with these expectations. Ironically perhaps, a photometric system based on apparent brightness will not conform to these *prima facie *expectations. As will be discussed in more detail in the next section, it is possible to show that when two lights of equal brightness are added together their sum can actually appear *less *bright than *either *light alone. Commerce and government simply could not employ a non-additive system of photometry where summing more optical radiation produced less light.

The significance of additivity in the definition, and thereby, the sale and regulation of light should not be underestimated. Not only does additivity ensure that the combination of optical radiation always increases the amount of measured light, additivity also provides for inexpensive and practical means of measuring that light. Additivity ensures that, at any level of optical radiation, a linear detector-filter combination matching the spectral response of V_λ _will provide photometric quantities identical to the sum of the spectral power obtained at each wavelength by a much more expensive and complicated spectroradiometer. Additivity is the dominant and perhaps only reason V_λ _has not been displaced by commerce and government after nearly a century of research showing the inherent limitations of V_λ _for characterizing the visual stimulus [16,18].

## Spectral sensitivity of brightness perception

In addition to the visual system's achromatic luminance channel, the spectral sensitivity of which is well characterized by V_λ_, two spectrally opponent color channels simultaneously contribute to our perceptions of brightness. The three visual channels leading to brightness perceptions are formed in the retina from the three cone photoreceptor types (L, M, and S cones) but, depending upon the subsequent neural connections, they are combined in different ways to provide brightness information to the visual cortex. As previously discussed, the spectral sensitivity of the luminance channel is dominated by the summed input from the L and M cones. The two color channels, red versus green (r-g) and blue versus yellow (b-y) are termed spectrally opponent channels because each provides opposing color information to the brain [19].

For one type of r-g channel, excitatory input is provided by the L cones and inhibitory input is provided by the M cones. For example, when the L cone provides relatively more input to the r-g channel than the M cone, the r-g channel signals "red" to the brain. Similarly, for one type of b-y channel, excitatory input is provided by the S cones and inhibitory input is provided by both the L cones and the M cones. When a light stimulates the S cones more than the combined input from the L and M cones, the b-y channel signals "blue" to the brain.

As spectrally opponent systems, these channels can signal *either *"red" or "green" and *either *"blue" or "yellow" to the brain. Moreover, a spectrally opponent system is inherently a subadditive system because the addition of, say, a "green" light to a "red" light can *decrease *the response of the r-g system cell. Since the two spectral opponent channels contribute to brightness perception, two lights added together can actually appear *less *bright than either light alone.

**Figure 2:**
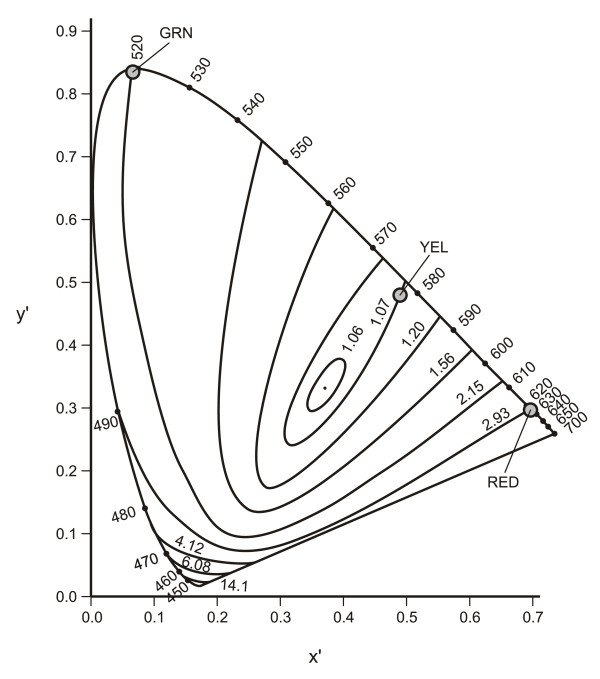
**Chromaticity diagram based on the Judd correction **[22], **showing contours of equal B/L value **[23]. Also shown are the chromaticity coordinates for a red light (RED) and for a green light (GRN) that, when added, produce a yellow light (YEL) with the illustrated chromaticity coordinates as described in the text.

Much of the research attempting to understand human brightness perception has utilized both photometry and colorimetry as indirect methods of measuring the apparent brightness of lights of different spectral compositions. Colorimetry originated from controlled observations in the 19^th ^century showing that with three, and only three, so called *primary *lights humans can match the appearance of any other test light [20]. In other words, by adjusting the radiances of the three primary lights, it was possible to create an additive mixture of these primary lights that was completely indistinguishable from the test light. (Some very saturated color stimuli cannot be matched using a set of three physical primaries without slightly changing the color of the test light by adding one of the physical primaries to it. Mathematically, this is equivalent to using a negative amount of primary to make the match. To avoid the use of negative amounts of primaries, the CIE color system [21] makes use of imaginary primaries that are mathematically defined, but not physically realizable.) As shown in subsequent research, the mixture matches the test light because the photon absorptions by the three cone photoreceptors are exactly the same for the mixture of primaries and for the test. Thus, the color of any test light can be quantified in terms of the relative amounts of the primary lights needed to match its appearance. The radiant powers in the three primaries are typically normalized with a linear transformation so that their sum is unity. By knowing two of the normalized values, the third value is also known. In this way the color, or more precisely the chromaticity, of the test light can be illustrated graphically in two dimensions. Figure 2 illustrates the results of colorimetric calculations based on the spectral power distribution of the light source and the three color matching functions in the (x', y') color system. (This color system is nearly identical to the Commission Internationale de l'Eclairage (x, y) 1931 color system presently in common use [21], but with small differences for short [<460 nm] wavelengths [22].) The physical specification of the chromaticity of any light, natural or fabricated, can be defined as a single point within the area enclosed by the outermost contour.

Of course, quantifying the relative amounts of three primaries needed to match the test light does not fully characterize its color appearance. A stimulus that is recognized as orange and another that is recognized as brown can have the same chromaticities, but factors such as the objects' relative luminances against their surrounding luminances will influence their perceived colors. Unlike photometry, where V_λ _closely characterizes the spectral sensitivity of the human fovea for some types of visual tasks [16], colorimetric specification of a test light does not represent the response of a single visual channel or even the combination of multiple visual channels. Since brightness is influenced by both chromatic as well as achromatic visual channels, additional techniques must be used to characterize and measure apparent brightness.

One common method of quantifying the brightness of lights is combining photometry and colorimetry by utilizing the ratio of the luminance needed by a test light of a given chromaticity to match the brightness of a reference light of another chromaticity and of a known luminance. The luminance of the reference light is designated B and the luminance of the test light that matches its brightness is designated L. Therefore, the brightness of any light in chromaticity space relative to a given reference light source can be described in terms of a unitless ratio, its B/L value. Figure 2 also shows constant B/L contours in the (x', y') color space [23] and, as described next, illustrates the subadditive nature of brightness perception.

Consider a red light (a 630-nm spectral light) and a green light (a 520-nm spectral light), with (x', y') chromaticity coordinates of (0.70, 0.30) and (0.07, 0.83), respectively, as shown in Figure 2. Suppose the luminance of the red light is 10 cd/m^2 ^and that of the green light is 15 cd/m^2^. Using the B/L values from Guth et al. [23] shown in Figure 2 (2.93 for red and 2.15 for green), their apparent brightnesses can be calculated from the product of their luminance (L, in cd/m^2^) and their B/L value:

• red: 10 cd/m^2 ^× 2.93 = 29.3

• green: 15 cd/m^2 ^× 2.15 = 32.3

If the red and the green lights are superimposed onto each other, the luminance of the resulting yellow light would, of course, be 25 cd/m^2 ^(10 + 15 cd/m^2^). The (x', y') chromaticity coordinates of this yellow light are (0.48, 0.49), corresponding to a B/L value of 1.07 using the B/L contours from Guth et al. [23] in Figure 2. Therefore, the apparent brightness of this yellow light can be calculated as it was for the red and green lights:

• yellow: 25 cd/m^2 ^× 1.07 = 26.8

Remarkably, the brightness of the yellow light created by combining the original red and green lights appears *less *bright than either the red or the green light alone, despite the yellow light being created from the superimposition of the red and green lights.

## A "photodian" luminous efficiency function

It seems natural that as more research is conducted on the impact of optical radiation on the circadian system, particularly as it might affect human health, attempts would be made to develop a spectral sensitivity function for the circadian system. It also seems natural that attempts would be made to develop an additive sprectral efficiency function comparable to V_λ _for the circadian system, a C_λ _[24,25]. Certainly it is possible to develop such a function from the available data (e.g., [26,27]) through international consensus to support commerce and government, but it is important to point out why an additive function like C_λ _could probably never be exactly comparable to V_λ_.

Intrinsically photosensitive retinal ganglion cells (ipRGCs) have been shown to provide direct input to the SCN [28,29]. Figueiro et al. [30] were the first to suggest that multiple photoreceptors contributed to human circadian phototransduction via color opponent processes distal to the ipRGCs in the retina. Spectral opponency is an inherent attribute of the human retina, initiated distal to the ipRGCs in the outer plexiform layer of the retina, and underlies both color perception and the subadditive nature of apparent brightness perception previously described. Demonstrations of subadditivity in human circadian phototransduction have been performed by Figueiro et al. [31] specifically designed to test the conclusions by Figueiro et al. [30]. More recently, Figueiro and colleagues demonstrated, as predicted, that the subadditive response to light by the circadian system is formed from spectral opponent mechanisms in the retina [32]. It is interesting in this regard that additivity has been demonstrated in mouse circadian phototransduction [33,34]. This species does not exhibit subadditivity presumably because, quite unlike humans, mice have very little neural apparatus to support color vision [35].

V_λ_, as previously discussed, is based upon a specific experimental paradigm isolating the achromatic visual channel in the fovea. This channel has been shown to be additive in response to optical radiation for a given criterion response (i.e., a constant-luminance flicker criterion). Our current understanding of the circadian system indicates that there is only one functional channel leading to the SCN from the retina, and that in humans, this channel exhibits subadditivity to certain combinations of wavelengths [36]. Clearly, a more detailed understanding of input to the SCN may emerge following additional research. For example, Aggelopolous and Meissl [37] suggest that there are multiple neural pathways providing input to the SCN in rats. Whether these different neuron pathways exist in humans or constitute different functional channels for the SCN has yet to be determined. Since there is no evidence to date that the human circadian system exhibits additivity, an additive "photodian" luminous efficiency function for measuring circadian light (i.e., a C_λ_) would only serve as a convenience to commerce and government. In other words, unlike V_λ_, there would be no physiological foundation for a system of metrology based upon C_λ_. This lack of homology between physiology and metrology may or may not be an important aspect in the deliberations for developing a system of circadian photometry, but it is certainly important to draw attention to this difference for scientific purposes, much as it is important to draw attention to the difference between luminance and brightness.

## Spectral sensitivity of the circadian system

The retino-hypothalamic tract (RHT) is comprised of ipRGC axons and carries photic information from the retina to the SCN. In addition to the direct conversion of optical radiation to neural signal input to the master clock, the ipRGCs also carry spectrally-opponent information originating from the classical photoreceptors and processed by vertical (bipolar cells) and lateral (horizontal and amacrine cells) neurons, to the SCN. Of particular interest with regard to developing a definition of circadian light are the spectrally-opponent (color) mechanisms in the distal retina that provide synaptic connections to the ipRGCs [32]. In addition, amacrine cells that control the transition from scotopic (rod) to photopic (cone) responses in retinal ganglion cells also appear to provide synaptic threshold control of the ipRGC responses. These complicated neural connections have been mathematically modeled to develop a definition of the circadian (light) stimulus [36]. The mathematical model of human circadian phototransduction developed by Rea et al. [36] is based on the neuroanatomy and neurophysiology of the retina and on published psychophysical studies of nocturnal melatonin suppression using lights of different spectral power distributions. The model generates values of circadian light (CL) for any spectral power distribution (i.e., for any light source, real or imagined, at any irradiance). CL is characterized by a high absolute threshold to optical radiation with a peak spectral response at short wavelengths. The model accounts for participation of ipRCGs as well as rods and cones in circadian phototransduction via neural connections, including spectral opponency, in the outer plexiform layer of the retina. Additional file 1 describes the computation procedure for calculating CL. It should be noted that the term CL is used in this paper to replace the term *circadian stimulus *(CS), used in the paper that describes the model of circadian phototransduction [36]. Notwithstanding the nonlinearities inherent in the circadian phototransduction model, CL is spectrally weighted irradiance for the human circadian system, a term more comparable to illuminance, which is spectrally weighted irradiance for the human visual system. As described in more detail below, the term CS will be henceforth used to describe the *effective *photic stimulus for the circadian system as measured by acute nocturnal melatonin suppression.

**Figure 3:**
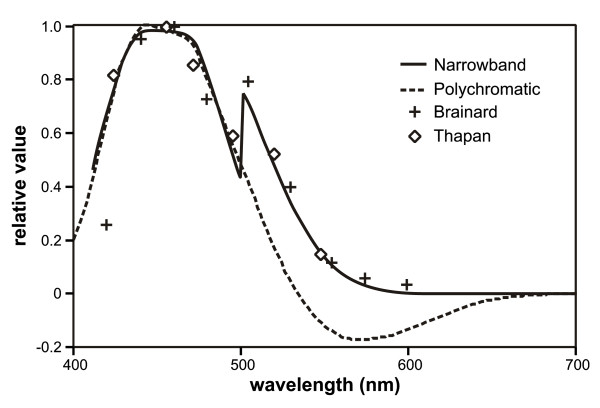
**Nocturnal human melatonin suppression data from Brainard et al**. [26]** and Thapan et al**. [27]** for narrowband spectra (symbols), and a spectral sensitivity function resulting from exposure to narrowband illumination (solid curve).** Also shown is the spectral sensitivity for broadband illumination when spectral opponency is exhibited [36].

**Figure 4:**
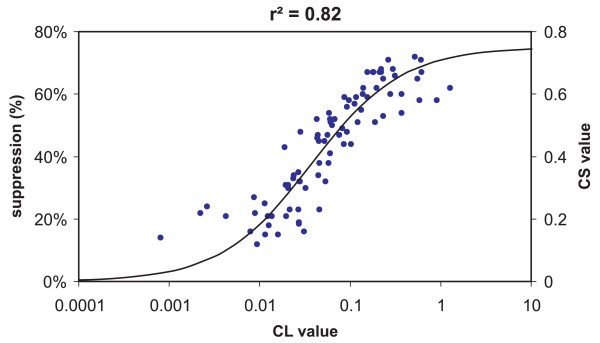
**Nocturnal human melatonin suppression data **[24,26,27,30,38,39]** (left ordinate), plotted as a function of CL quantities (abscissa) predicted by the model of Rea et al**. [36]. Also shown is the best-fitting four-parameter logistic function [40] to all of the data from threshold to saturation. The circadian light quantity CL was formerly named circadian stimulus (CS) [36]; CS (right ordinate) now refers to the effective stimulus based on nocturnal melatonin suppression.

Figure 3 shows the modeled spectral sensitivity of the circadian system for both narrowband and polychromatic light stimuli [36]. Because the model includes spectral opponency, responses from light stimuli created by a combination of narrowband sources cannot be predicted from the spectral sensitivity derived from narrowband light stimuli alone. In fact, for light stimuli with a particular balance of short-wavelength (e.g., around 450 nm) and long-wavelength (e.g., longer than about 510 nm) components, the response of the human circadian system to light is less than what would be predicted by an additive spectral efficiency function derived from responses to narrowband stimuli [30,31]. Emphasis for modeling was placed upon studies measuring nocturnal melatonin suppression because, in fact, there are presently no comparable spectral sensitivity functions for the circadian system using any other outcome measure (e.g., phase shifting). The values of the coefficients in Additional file 1 relating the opponent channels were optimized to align with published nocturnal human melatonin suppression data using narrowband spectra [26,27]. This resulted in a good fit (r^2 ^= 0.82) between all comparable suppression data using both narrowband and broadband spectra [24,26,27,30,38,39] and a four-parameter logistic function [40] characterizing the melatonin suppression response as the light stimulus increases from threshold to saturation (Figure 4). CL is defined in terms of irradiance, not radiance as with brightness because image formation on the retina is not believed to be important to the circadian system. Rather, CL is geometrically described in terms of radiant flux density on the cornea and therefore is geometrically comparable to illuminance at the eye.

Previously published studies have been conducted to test the utility of the model of human circadian phototransduction [32,41]. Nocturnal melatonin suppression by light was assessed by Figueiro et al. [41] for two light source spectra and four light levels. *A priori *predictions of melatonin suppression were made based on calculations of CL given by each light level and spectra. Results demonstrated that the model predictions were consistent with melatonin suppression obtained at all four light levels, although uncertainty was greater at the lowest light level, which was close to threshold response. Also, Figueiro et al. [32] measured nocturnal melatonin suppression following exposure to lights presented monocularly and binocularly to demonstrate that the subadditive response to light by the circadian system originated in the retina as predicted by Rea et al. [36].

Figueiro et al. [42] measured nocturnal melatonin suppression from short-wavelength light stimuli; these data were consistent with predictions made using the model by Rea and colleagues [36]. Similarly, noctural melatonin suppression measurements reported by Revell and Skene [43] in response to narrowband and broadband light stimuli varying in intensity were shown [44] to be consistent with predictions using this model [36].

## Utilization of circadian light

As previously described, Rea et al. [36] proposed a mathematical model for quantifying circadian light for any spectral irradiance distribution. Two changes in the circadian light nomenclature from that paper have been made for metrological clarity. First, because the units of CL (spectrally weighted irradiance in W/m^2^) are new and, therefore, are not particularly intuitive to a user, a normalized quantity, CL_A_, was derived to more easily compare CL values with values of photopic illuminance, in lux (lx). A value of CL can be determined, measured or calculated, for 1000 lx of CIE standard illuminant A [21], a blackbody radiator at a color temperature of 2856 K similar in relative spectral power distribution to an incandescent lamp, and a scalar multiplier determined to make the product of CL and the multiplier equal 1000. The product of CL and this multiplier defines the quantity CL_A_. Any value of CL can then be normalized in terms of a reference illuminance of 1000 lx from the standard illuminant A equaling 1000 CL_A _units. CL_A _is numerically identical to orthodox photopic illuminance when illuminant A produces 1000 lx, but can differ, sometimes significantly, for other spectral power distributions and illuminance levels due to nonlinear operations in the CL formulation (see Additional file 1). Nevertheless, for many common white light sources values of CL_A _are similar in magnitude to illuminance values (in lx) at any level.

Second, *circadian stimulus *(CS; [3,36,44,45]) in the original formulation is now defined as CL and, after normalization, as CL_A_. To understand why, consider two light sources producing very different irradiance and spectral quantities, resulting in CL_A _values of 10,000 and of 20,000 units. Despite a large difference in the values of CL_A_, the two sources would not be expected to produce different outcomes from the circadian system, at least in terms of nocturnal melatonin suppression. Both would produce saturating levels of suppression of about 75% percent after an hour of exposure. Thus, while the two sources would be characterized as being very different in terms of CL_A_, their effectiveness as a circadian stimulus in terms of nocturnal melatonin suppression would be identical. The term CS for a given light source is therefore now defined in terms of the *relative effectiveness *of CL, or CL_A_, for producing a meaningful circadian response. The logistic function in Figure 4 is used to relate a given CL, or CL_A_, value to its corresponding CS value, between 0 (or 0%) and 0.75 (or 75%), characterizing the relative effectiveness of the source as a stimulus to the circadian system.

The implications for establishing quantitative measures of CL, CL_A _and CS are key to developing an understanding of how temporal patterns of light and dark affect human health and well-being. Without a quantitative understanding of the circadian light stimulus it will be difficult or impossible to make significant progress in unraveling the role that circadian disruption has on diseases such as breast cancer [2,3], cardiovascular disease [4-6], diabetes [7,8] and sleep disorders [9].

**Figure 5:**
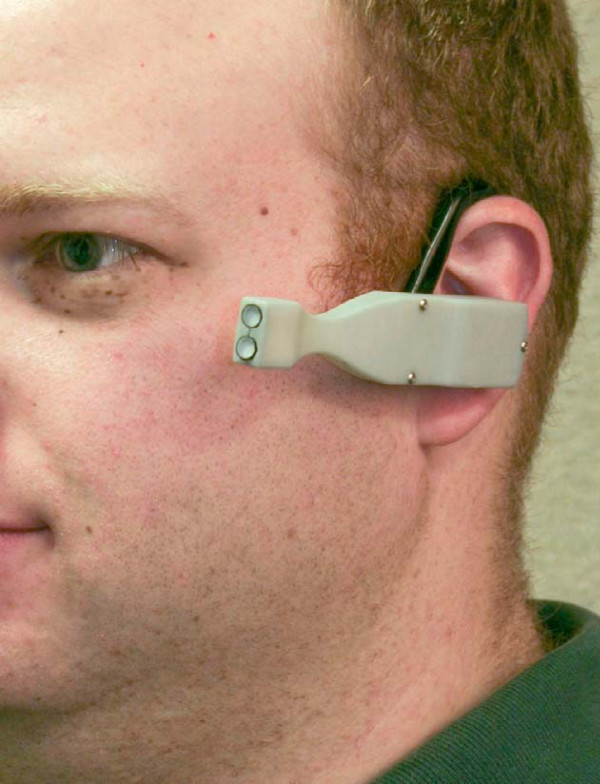
**Close-up photograph of the Daysimeter**. Two light sensors are located near the plane of the cornea, calibrated in terms of their absolute, spatial and spectral response to provide both photopic and circadian light readings. The rear housing attached to the earpiece contains accelerometers for measuring activity as well as memory and control circuitry, all powered by a coin-cell battery.

Toward this end a circadian light dosimeter, the Daysimeter (Figure 5), was developed to quantify circadian light exposures in these vulnerable populations. The Daysimeter, previously described [46] and subsequently refined [47], is a personal head-worn device that measures CL_A _and photopic illuminance near the plane of the wearer's cornea. The Daysimeter also includes calibrated accelerometers to measure rest and activity. Data from the Daysimeter are recorded for as long as one month of wear and retrieved for post-processing. Each Daysimeter has its own spectral, spatial, and absolute light calibration so, following post-processing, it is possible to quantify individual CL_A _exposures in real life over extended periods. These data have great potential for understanding the impact of circadian disruption on human health because, for the first time, researchers and clinicians can actually measure circadian disruption among individuals in these vulnerable populations. Although beyond the scope of this paper, the Daysimeter has, in fact, recently been used to quantify and compare circadian disruption in day-shift and rotating-shift nurses [47,48]. Future research will undoubtedly utilize instruments like the Daysimeter to develop, for example, new shift-work schedules, new architectural practices and new light sources, all of which will depend upon our collective ability to measure and calculate circadian light.

## Circadian light as a work in progress

The definition of circadian light proposed here is based on the current knowledge of the neuroanatomy and neurophysiology of the human retina and on published psychophysical studies of nocturnal melatonin suppression using lights of different spectral power distributions. CL_A _and CS are provisionally defined then in terms of what is known about nocturnal melatonin suppression in humans (for an hour-long exposure to light, near the midpoint of the melatonin production curve, and with naturally-constricted pupils). Of course, acute melatonin suppression is not the only non-visual response by the circadian system; other non-visual responses include phase shifting and alertness. For at least these two cases, however, light-induced phase-shifting and light-induced nocturnal alertness appear to have similar threshold-to-saturation response characteristics [40,44]. A very recent study, however, has shown that both red and blue lights can affect alertness [49] as well as cortisol and alpha amylase production (Figueiro and Rea., unpublished data) at night indicating that not all light-induced, non-visual responses have the same spectral sensitivity as nocturnal melatonin suppression. The development of new response characteristics for these non-visual systems, if they emerge, would be very reminiscent of those that were developed in visual science where multiple spectral sensitivity functions for different visual channels were established (cf. Figure 1). If it is shown that the relationships between CL_A _and other non-visual responses, such as phase shifting, are different than the one demonstrated for nocturnal melatonin suppression, another CS function could be developed and designated with an appropriate subscript (such as CS_nmel _for nocturnal melatonin suppression and CS_pshift _for phase shifting). Again, this development would be quite similar to the evolution of different visual spectral sensitivity functions.

As a final note, even the model of human circadian phototransduction based upon nocturnal melatonin suppression and used to calculate CL_A _and CS is probably incomplete. It does not take into account possible participation of different types of ipRGCs [50] and recent evidence that the melanopsin photopigment in the ipRGCs follows a very different regenerative process than that employed by the classical photoreceptors [51]. These phenomena may have heretofore unknown effects on the spectral and absolute sensitivities of the circadian system that would demand consideration in a revised model of phototransduction and therefore an evolving definition of circadian light. Hopefully, however, the information presented here is an important step toward the precise application of light stimuli for the human circadian system.

## Conclusions

Light is formally defined as optical radiation capable of providing visual sensation in humans. The current definition of light does not directly relate to its effects on the human circadian system. Since temporal patterns of retinal light (and dark) exposures regulate the human circadian system and since disruption of the circadian system has broad implications for health and well-being [2-9,52,53], it is becoming increasingly important to develop a new definition of circadian light.

Toward that end, the present paper has placed the evolving development of a definition of circadian light into the historical context of light as it has been defined for metrology and as it affects human vision. As described here, an additive "photodian" luminous efficiency function for circadian light will probably never be exactly comparable to the photopic luminous efficiency function used in conventional photometry based upon the human visual system. Nevertheless, it is increasingly important that a measurement system, such as CL, CL_A_, and CS as presented here, be developed for quantifying the photic stimulus for the human circadian system.

## Competing interests

The authors declare that they have no competing interests.

## Authors' contributions

The outline of the article was developed by all co-authors. MSR led the effort and wrote a partial draft of the manuscript with MGF. AB and JDB wrote specific sections of the text and prepared the figures. All co-authors reviewed and approved the final manuscript.

## Supplementary Material

Additional file 1 **Circadian light (CL, CL_A_) and circadian stimulus (CS) calculation procedure **[10,21,36,54-56]. Click here for file
