# Supplementary material for: Circadian light
Source: J Circadian Rhythms. 2010 Feb 13;8:2. doi: 10.1186/1740-3391-8-2 (PMC2851666; doi:10.1186/1740-3391-8-2)

Additional file 1: Circadian light (CL, CL$_A$) and circadian stimulus (CS) calculation procedure

The following equations define CL, and are shown in a similar format as originally published [36; all citation numbers in this file refer to references listed in the main paper]. Additional equations for CL$_A$ and CS follow.

$$CL = \left[\left(a_1 \int P_\lambda M_\lambda d\lambda - b_1\right) + a_2\left(\int P_\lambda S_\lambda d\lambda - k\int P_\lambda V_{10\lambda} d\lambda\right) - b_2\right] - a_3\left(1 - e^{-\left(\frac{\int P_\lambda V'_\lambda d\lambda}{rodSat}\right)}\right)$$

for $\int P_\lambda S_\lambda d\lambda - k\int P_\lambda V_{10\lambda} d\lambda \geq 0$ (Eq. A1a)

$$CL = a_1 \int P_\lambda M_\lambda d\lambda - b_1$$

for $\int P_\lambda S_\lambda d\lambda - k\int P_\lambda V_{10\lambda} d\lambda < 0$ (Eq. A1b)

where:

M_λ is the melanopsin-containing retinal ganglion cell spectral efficiency function peaking at 480 nm [54],
$V_{10\lambda}$ is the large-field L+M cone spectral efficiency function [55],
V'_λ is the rod spectral efficiency function [10],
S_λ is the S cone spectral efficiency function [56],
P_λ is the spectral irradiance at the eye ($W/m^2/nm$),

Parameters represent the interactions among photoreceptor types
$k = 0.31$
$a_1 = 0.285$
$a_2 = 0.2$
$a_3 = 0.72$

Constants represent the thresholds and dynamic characteristics of the photoreceptor types as described below
$b_1 = 0.01$
$b_2 = 0.001$
$rod_{Sat} = 6.5$
CL (circadian light) is in units of circadian spectrally weighted irradiance (weighted W/m^2).

In the model, when the b-y channel signals “blue”, depolarization for a positive value, the computed response is added to the computed ipRGC depolarization response (Equation A1a); however, when the b-y channel signals “yellow”, hyperpolarization for a negative value, only the computed ipRGC depolarization response contributes to CL (Equation A1b).

Step-by-step calculations

Step 1: Calculate the four photoreceptor responses used in the model

To determine CL, four photoreceptor responses to optical radiation are needed. The rod response is based on the scotopic luminous efficiency function, V'_λ, and is denoted by R. In the model rods limit the effective signaling of the other photoreceptors, but with diminishing effect at higher irradiance levels as rods are bleached. The ipRGC response is defined in terms of the melanopsin response function, denoted by G. ipRGCs provide direct input to the SCN via the RHT. The b-y spectral opponent mechanism providing direct input to the ipRGCs is formed by the S cone response, denoted by B, and $V_{10\lambda}$, the sum of the L cone and M cone responses, denoted by Y. In practice, because both the spectral power distribution of the stimulus, P_λ, and the relative photoreceptor spectral response functions are not expressed as functions, but rather as measured quantities with the values given in discrete, tabular form, the integrals are replaced with summations.

$$R = \sum_{\lambda=380}^{730} P_\lambda V'_\lambda \Delta\lambda \qquad \text{(Eq. A2)}$$

$$G = \sum_{\lambda=380}^{730} P_\lambda M_\lambda \Delta\lambda \qquad \text{(Eq. A3)}$$

$$B = \sum_{\lambda=380}^{730} P_\lambda S_\lambda \Delta\lambda \qquad \text{(Eq. A4)}$$

$$Y = k \sum_{\lambda=380}^{730} P_\lambda V_{10\lambda} \Delta\lambda \qquad \text{(Eq. A5)}$$

Note that the Y response is scaled by the parameter *k* in Equation A5. The summations must extend over the range of wavelengths where the product of the stimulus spectral power

distribution (P_λ) and the photoreceptor response functions are non-zero. Practically, wavelengths shorter than 380 nm or longer than 730 nm are almost always of negligible significance to the CL calculation. Interpolation of the response values is often necessary so as to match the wavelength increments of the four response functions to the tabulated SPD values. Figure A1 shows the four response functions used in the CL calculation. S_λ is the S cone fundamental [56] underlying B in the CL equation; V'_λ is the scotopic luminous efficiency function [10] underlying R in the CL equation; M_λ is the melanopsin spectral response function [54] underlying G in the CL equation; $V_{10\lambda}$ is the ten-degree photopic luminous efficiency function [55] underlying Y in the CL equation. Table A1 tabulates the values for these four response functions in 10-nm increments.

Table A1. Spectral response functions for the model, tabulated in 10-nm increments from 380 to 730 nm.

**Wavelength** (nm)	$\mathbf{S}_\lambda$ (S cone)	$\mathbf{V'}_\lambda$ (Scotopic)	$\mathbf{M}_\lambda$ (ipRGC)	$\mathbf{V}_{10\lambda}$ (Photopic)
380	0.0000	0.0000	0.0000	0.0000
390	0.0078	0.0022	0.0668	0.0003
400	0.1740	0.0093	0.1541	0.0020
410	0.3629	0.0348	0.2755	0.0088
420	0.6612	0.0966	0.4060	0.0214
430	0.9044	0.1998	0.5240	0.0387
440	1.0000	0.3281	0.6310	0.0621
450	0.9161	0.4550	0.7450	0.0895
460	0.8019	0.5670	0.8620	0.1282
470	0.6931	0.6760	0.9570	0.1852
480	0.4701	0.7930	1.0000	0.2536
490	0.2774	0.9040	0.9680	0.3391
500	0.1647	0.9820	0.8670	0.4608
510	0.0956	0.9970	0.7170	0.6067
520	0.0474	0.9350	0.5480	0.7618
530	0.0256	0.8110	0.3864	0.8752
540	0.0124	0.6500	0.2519	0.9620
550	0.0054	0.4810	0.1517	0.9918
560	0.0023	0.3288	0.0845	0.9971
570	0.0016	0.2076	0.0438	0.9555
580	0.0008	0.1212	0.0215	0.8689
590	0.0008	0.0655	0.0101	0.7774
600	0.0008	0.0331	0.0046	0.6583
610	0.0000	0.0159	0.0021	0.5280
620	0.0000	0.0074	0.0009	0.3981
630	0.0000	0.0033	0.0004	0.2835
640	0.0000	0.0015	0.0002	0.1798
650	0.0000	0.0007	0.0001	0.1076
660	0.0000	0.0003	0.0000	0.0603
670	0.0000	0.0001	0.0000	0.0318
680	0.0000	0.0001	0.0000	0.0159
690	0.0000	0.0000	0.0000	0.0077
700	0.0000	0.0000	0.0000	0.0037
710	0.0000	0.0000	0.0000	0.0018
720	0.0000	0.0000	0.0000	0.0008
730	0.0000	0.0000	0.0000	0.0004

Step 2: Determine whether the b-y spectral response function provides input into the model.

Subtract the Y response from the B response; if the remainder is negative, then the b-y spectral opponent response does not contribute to CL. Proceed to step 4; else, continue with step 3.

Step 3: Determine rod shunting

If the b-y spectral opponent response is not negative, the rod response controls the level of CL by an amount dependent on the rod response. As the level of optical radiation increases the rod system saturates and loses control of the overall response. Rod saturation is modeled as:

$$R_{shunt} = 1 - e^{-\left(\frac{R}{rodSat}\right)}, \quad \text{(Eq. A6)}$$

where *rodSat* is the rod saturation constant, equal to 6.5.

Step 4: Combine photoreceptor channels

If the b-y spectral opponent response is non-negative [i.e., (B-Y)≥0]:

$$CL = \left[\left(a_1 G - b_1\right) + \left(a_2\left(B - Y\right) - b_2\right)\right] - a_3 R_{shunt} \quad \text{(Eq. A7)}$$

Else if (B-Y)<0,

$$CL = a_1 G - b_1 \quad \text{(Eq. A8)}$$

Negative values are possible, occurring for low irradiance values, and are interpreted as the CL being below threshold and effectively zero. The negative values result from the parsimonious manner in which the threshold for each response function is modeled as a linear response with an offset value (b_1 and b_2). Therefore,
if $CL < 0$, $CL = 0$. (Eq. A9)

Step 5: Normalize CL value

$$CL_A = 5831 CL \quad \text{(Eq. A10)}$$

CL_A values are normalized so that a stimulus with a spectral power distribution defined by CIE [21] Illuminant A (a blackbody radiator at 2856 K, similar to an incandescent lamp) having an photopic illuminance at the cornea of 1000 lx equals a CL_A value of 1000. The normalization relates CL to the SI unit of illuminance and provides a convenient and familiar reference value for better interpreting CL magnitudes. Generally speaking, light sources having high correlated color temperatures (e.g., daylight, around 5000 K or higher) will have CL_A values greater than the corresponding photopic illuminance value and light sources with low correlated color temperatures (e.g., fire light, around 2000 K or lower) will have CL_A values lower than the corresponding photopic illuminance value. In addition, stimuli at illuminance values less than 1000 lx will have CL_A values lower than the corresponding photopic illuminance due to non-linear effects of rod shunting (R_{shunt}) in the model.

Step 6: Determine resulting CS value

$$CS = 0.75 - \frac{0.75}{1+\left(\frac{CL_A}{215.75}\right)^{0.864}} \qquad \text{(Eq. A11)}$$

Example

Below is an example of the calculation of CL, CL_A and CS for an illuminance of 1000 lx at the eye from daylight (CIE D_{65} [21]) illumination.

Step 1. Calculate the four photoreceptor responses used in the model

	**P**	**R**	**G**	**B**	**Y**
Wavelength (nm)	Irradiance (W/m^2)	$P_\lambda * V'_\lambda * \Delta\lambda$	$P_\lambda * M_\lambda * \Delta\lambda$	$P_\lambda * S_\lambda * \Delta\lambda$	$k * P_\lambda * V_{10\lambda} * \Delta\lambda$
380	0.0069	0.0000	0.0000	0.0000	0.0000
390	0.0076	0.0002	0.0051	0.0006	0.0000
400	0.0115	0.0011	0.0177	0.0200	0.0001
410	0.0127	0.0044	0.0350	0.0461	0.0003
420	0.0129	0.0125	0.0524	0.0853	0.0009
430	0.0120	0.0240	0.0629	0.1085	0.0014
440	0.0145	0.0476	0.0915	0.1450	0.0028
450	0.0162	0.0737	0.1207	0.1484	0.0045
460	0.0163	0.0924	0.1405	0.1307	0.0065
470	0.0159	0.1075	0.1522	0.1102	0.0091
480	0.0161	0.1277	0.1610	0.0757	0.0127
490	0.0151	0.1365	0.1462	0.0419	0.0159
500	0.0151	0.1483	0.1309	0.0249	0.0216
510	0.0149	0.1486	0.1068	0.0142	0.0280
520	0.0145	0.1356	0.0795	0.0069	0.0342
530	0.0149	0.1208	0.0576	0.0038	0.0404
540	0.0145	0.0943	0.0365	0.0018	0.0432
550	0.0144	0.0693	0.0218	0.0008	0.0443
560	0.0139	0.0457	0.0117	0.0003	0.0430
570	0.0133	0.0276	0.0058	0.0002	0.0394
580	0.0133	0.0161	0.0029	0.0001	0.0358
590	0.0123	0.0081	0.0012	0.0001	0.0296
600	0.0125	0.0041	0.0006	0.0001	0.0255
610	0.0124	0.0020	0.0003	0.0000	0.0203
620	0.0122	0.0009	0.0001	0.0000	0.0151
630	0.0115	0.0004	0.0000	0.0000	0.0101
640	0.0116	0.0002	0.0000	0.0000	0.0065
650	0.0111	0.0001	0.0000	0.0000	0.0037
660	0.0111	0.0000	0.0000	0.0000	0.0021
670	0.0114	0.0000	0.0000	0.0000	0.0011

680	0.0108	0.0000	0.0000	0.0000	0.0005
690	0.0097	0.0000	0.0000	0.0000	0.0002
700	0.0099	0.0000	0.0000	0.0000	0.0001
710	0.0103	0.0000	0.0000	0.0000	0.0001
720	0.0085	0.0000	0.0000	0.0000	0.0000
730	0.0097	0.0000	0.0000	0.0000	0.0000
**Summation totals**		**1.4494**	**1.4409**	**0.9656**	**0.4991**

Step 2. Determine whether the b-y spectral response function provides input into the model

$$B - Y = 0.9656 - 0.4991$$

$$= 0.4665 \quad \text{(not negative, therefore opponency is active)}$$

Step 3. Determine rod shunting

$$R_{shunt} = 1 - e^{-\left(\frac{R}{rodSat}\right)}$$

$$= 1 - e^{-\left(\frac{1.4494}{6.5}\right)}$$

$$= 0.1999$$

Step 4. Combine photoreceptor channels

$$CL = \left[a_1 G - b_1 + a_2 (B - Y) - b_2\right] - a_3 R_{shunt}$$

$$= \left[0.285 \times 1.4409 - 0.01 + 0.2 \times (0.9656 - 0.4991) - 0.001\right] - 0.72 \times 0.1999$$

$$= 0.3490$$

Step 5. Normalize CL value

$$CL_A = 5831 CL$$

$$= 5831 \times 0.3490$$

$$= 2035$$

Step 6. Determine resulting CS value

$$CS = 0.75 - \frac{0.75}{1 + \left(\frac{CL_A}{215.75}\right)^{0.864}}$$

$$= 0.75 - \frac{0.75}{1 + \left(\frac{2035}{215.75}\right)^{0.864}}$$

$$= 0.656 \qquad \text{(65.6\% suppression)}$$

Figure A1. Spectral response functions contributing to CL: S_λ, V'_λ, M_λ, $V_{10\lambda}$

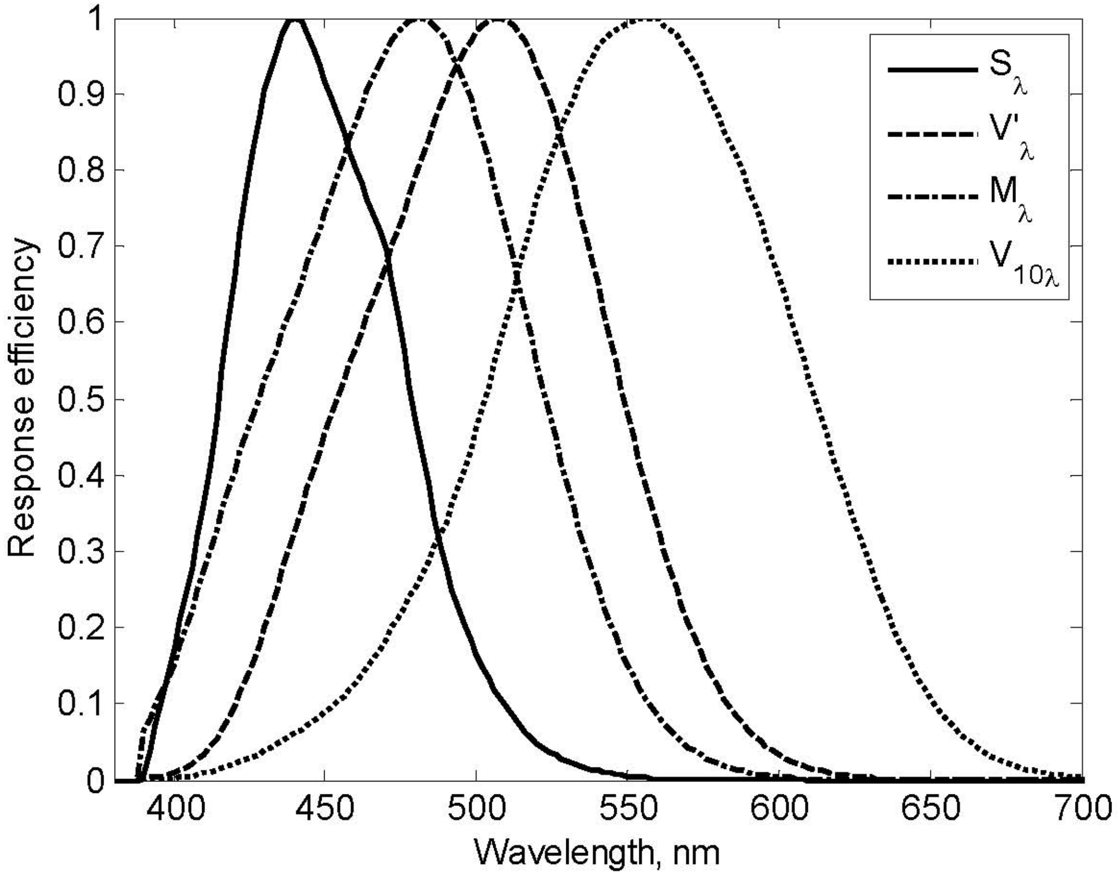

Supplement: Additional file 1 — Circadian light (CL, CLA) and circadian stimulus (CS) calculation procedure [10,21,36,54-56]. [file 1740-3391-8-2-S1.PDF]
